# Noninvasive Ventilation and Rapid Enteral Feeding Advances in Preterm Infants—2-Year Follow-Up of the STENA-Cohort

**DOI:** 10.3390/nu15051292

**Published:** 2023-03-06

**Authors:** Judith Behnke, Vanessa Estreich, Frank Oehmke, Bernd Axel Neubauer, Anita Windhorst, Harald Ehrhardt

**Affiliations:** 1 Department General Pediatrics and Neonatology, Justus Liebig University and Universities of Giessen and Marburg Lung Center, 35392 Giessen, Germany; 2 German Center for Lung Research (DZL), 35392 Giessen, Germany; 3 Department of Radiology, Institute for Diagnostic and Interventional Radiology, Justus Liebig University of Giessen, 35392 Giessen, Germany; 4 Department of Gynecology and Obstetrics, Justus Liebig University of Giessen, 35392 Giessen, Germany; 5 Department of Neuropediatrics, Justus Liebig University of Giessen, 35392 Giessen, Germany; 6 Department of Medical Statistics, Justus Liebig University of Giessen, 35392 Giessen, Germany; 7 Department of Pediatrics and Adolescent Medicine, Division of Neonatology and Pediatric Intensive Care Medicine, University Medical Center Ulm, 89075 Ulm, Germany

**Keywords:** enteral feeding advances, nutrition, very low birthweight infants, noninvasive ventilation, neurodevelopmental outcome

## Abstract

The importance of nutritional supply for somatic growth and neurodevelopmental outcome in very-low-birthweight infants is an established medical strategy for reducing long-term morbidities. Our cohort study on rapid enteral feeding advances using a standardized protocol (STENA) previously demonstrated a 4-day reduction of parenteral nutrition. STENA did not impede the success of noninvasive ventilations strategies but significantly less infants required mechanical ventilation. Most importantly, STENA resulted in improved somatic growth at 36 weeks of gestation. Here, we evaluated our cohort for psychomotor outcomes and somatic growth at 2 years of age. *n* = 218 infants of the original cohort were followed-up (74.4%). Z-scores for weight and length did not differ but the benefits of STENA for head circumference persisted until the age of 2 years (*p* = 0.034). Concerning the psychomotor outcome, we neither found any statistically significant differences in the mental developmental index (MDI) (*p* = 0.738), norin the psychomotor developmental index (PDI) (*p* = 0.122). In conclusion, our data adds important insights on the topic of rapid enteral feeding advances and confirms the safety of STENA with respect to somatic growth and psychomotor outcome measures.

## 1. Introduction

The importance of adequate nutritional supply for somatic growth and neurodevelopmental outcome in very-low-birthweight infants (VLBWIs) is well acknowledged and provides a feasible strategy for reducing long-term morbidities following premature delivery [1,2,3,4,5,6]. Enhanced energy and macronutrient intake during the first four weeks of life have been associated with improved somatic growth, as well as with improved cortical development in the neonatal period and in long-term neurodevelopmental outcomes [1,7]. Moreover, postnatal undernutrition is an independent predictor of chronic lung disease [8]. Most evidence is available for the association of adequate nutritional supply and psychomotor outcomes in the preterm infant. Lately, the advantages for other organs including the immature lung got more and more acknowledged that needs to accomplish critical steps of lung development outside the uterus. The evidence is established that not only the provision of adequate caloric intake is a prerogative for an optimal outcome but the quality of nutritional supply is of utmost importance in this context [9,10,11,12]. During the recent years the benefits of breast milk supply became more and more evident which accelerates the aim of reaching rapid full enteral nutrition after delivery and reduces the risk of severe complications including late onset sepsis, focal intestinal perforation (FIP), necrotizing enterocolitis (NEC) and bronchopulmonary dysplasia which pose major threads to the preterm infant [13,14,15,16]. Several different nutrients have been specifically associated with brain structure and neurodevelopment. Deficits of these nutrients can affect the developing brain and especially early organizational events and major brain processes such as neurogenesis, cell migration and differentiation, myelination and synaptogenesis. While all nutrients are important for brain development, certain ones have particularly large influence in early preterm brain development: glucose as the primary energy source for the brain, macronutrients like protein and fats (including long-chain polyunsaturated fatty acids), as well as the micronutrients iron, zinc, copper, iodine, folate and choline [1,17,18,19]. The recently published position paper on enteral nutrition in preterm infants born <1800 g birthweight (BW) by the the European Society of Pediatric Gastroenterology, Hepatology and Nutrition (ESPGHAN) Committee of Nutrition (CoN) just updated the expert consensus on the optimum daily energy and macro- and micronutrition intake but as stated by the authors plenty of uncertainties remain and urgently demand increased knowledge, especially in focus on all immature organ systems of the preterm infant [20]. One further aspect that came into the focus of researchers during the last decade is the advancement of enteral feeding until full enteral feeds are accomplished [21,22]. In this context, rapid advances in enteral nutritional supply have long-time been questioned in preterm infants due to the major concerns of intestinal intolerance. Major concerns were particularly raised by experts with respect to the severe complications of FIP and NEC, and to increased respiratory instability due to abdominal distension. Those complications may also change the enteral feeding trajectory differently in preterm infants at different gestational age (GA) [6]. Preterm infants who develop NEC have a higher incidence of long-term neurological disability [23]. Of note, infants who require surgery for NEC have an even higher risk of poor outcome than those who receive only medical treatment. If this is a consequence of the infectious process itself or undernutrition during a critical period of brain development or a combination of both is uncertain [24]. The actual Cochrane meta-analysis of all randomized and quasi randomized controlled trials on this topic indicates no negative effects of rapid versus slow feeding advances on the risk for severe morbidities including NEC and FIP or all-cause mortality but may increase the risk of feeding intolerance and of late onset sepsis [25]. Most of the patients included into the meta-analysis were available from the only multicenter randomized trial on this topic and the only study investigating the neurodevelopmental outcome at 24 months of age. Of clinical importance, the SIFT study (*n* = 2470) did not detect any differences in survival without moderate or severe neurodevelopmental disability between slow and rapid enteral feeding advances [25,26]. Trial definition of moderate or severe neurodevelopmental disability was any of the following: moderate or severe visual impairment (reduced vision uncorrected with aids, blindness in one eye with good vision in the contralateral eye, or blindness or light perception only), moderate or severe hearing impairment (hearing loss corrected with aids, some hearing loss uncorrected by aids, or deafness), moderate or severe gross motor impairment (inability to walk or sit independently), or moderate or severe cognitive impairment as assessed with the use of the Parent Report of Children’s Abilities–Revised (PARCA-R) or clinical data if PARCA-R scores were missing [26]. Furthermore, one actual systematic review states that fast enteral feeding advances may also reduce the risk of apnea (RR 0.72, 95% CI 0.47 to 1.12, 2 trials, *n* = 153) and the duration of hospitalization (mean difference −3.08 days, 95% CI −4.34 to −1.81, 7 trials, *n* = 3864) [27]. The recent ESPGHAN publication concludes that minimal enteral feeding has no beneficial effect compared to advancing feeds immediately after birth and recommends starting small volume enteral feeds as soon as possible, as well as advancing feeds as clinically tolerated. With regard to enteral feeding advancement the CoN recommendation for stable preterm infants is a routine daily increment of 18–30 mL/kg/d, especially in breastmilk-fed infants provided that the clinician considers that feed volume can be increased [20]. Concerning growth standards to avoid growth faltering (GF) in high-risk preterm infants the position paper suggests a typical acceptable initial weight loss of 7–10%, reaching a nadir at days 3–4. Regaining of BW should be aimed by 7–10 days of age, followed by growth along a target centile and a gradual transition to the corresponding birth percentile on the World Health Organization (WHO) postnatal growth chart within the first weeks or months post term. Furthermore, nutritional management and growth assessment should not differ between infants born appropriate for gestational age (AGA) and small for gestational age (SGA). The authors conclude that if GF is recognized within recommended intake ranges, an accurate assessment whether undernutrition risks neurocognitive impairment or rapid catch-up growth causes adverse metabolic programming is required [20].

Another point of discussion is whether or not to evaluate gastric residuals before application of the next feeding volume. Prefeeding gastric residual evaluation has been introduced into clinical routine to identify clinical precursors of NEC and other severe morbidities early on. The actual Cochrane meta-analysis comprising two randomized controlled trials as well as a more recent systematic review and meta-analysis of 6 randomized studies on this topic both concluded that routine prefeed gastric residual aspiration does not reduce the incidence of NEC but is associated with a prolonged duration until full enteral feeds are reached [28,29]. Of clinical relevance, this clinical management does result in prolonged duration of parenteral nutrition and until full enteral feeds are reached this poses a relevantly increased risk for nosocomial infection to the vulnerable patient population. But it might even lead to a growth disadvantage that goes far beyond the later time point of regaining birth weight and the total duration of hospitalization [28,29]. One recent publication added data supporting the association of rapid enteral feeding advances with improved somatic growth of height and head circumference [30]. Potential explanations for this discrepancy come from further studies on this topic where in rapid enteral feeding advances were associated with less episodes of abdominal distension arguing towards better tolerability in enteral fees that enabled higher enteral intakes in the stable phase of postnatal growth [31]. One further aspect might arise from the quality and form of nutritional supply and it is not surprising that higher enteral feeds with a balanced nutrition and comparable total caloric intake lead to improved somatic growth as has been documented for lung growth and the diagnosis of bronchopulmonary dysplasia before [10]. Furthermore, gastric residuals vary depending on the position of the preterm infant and on the form of nutritional supply like intermittent bolus or continuous feeding [32,33].

The limitation of all the previous publications on this topic remains the heterogeneity between studies and the mostly small sample size, the heterogeneous background of high- and middle-income countries and the type of feeding applied that was not separated for breast and formula feeding [25,27]. Furthermore, all these former studies did not address the effects of rapid enteral feeding advances on somatic growth until discharge or even beyond and they did not reflect the feasibility of this measure in the context of keeping the preterm infant stable on noninvasive respiratory support which constitutes another treatment priority with the aim to avoid the deleterious effects of invasive mechanical ventilation. The relevance is confirmed by latest meta-analyses on this topic that display the relevance of avoiding mechanical ventilation but as well oxygen toxicity to the immature lung. In detail, the successful stabilization of preterm infants on noninvasive respiratory support and the risk of a poor pulmonary outcome and bronchopulmonary dysplasia (BPD) depends on many factors that include the baseline parameters gestational age and birth weight, gender and maternal disorders and habitats [34]. Furthermore, the avoidance of mechanical ventilation and thereby of shear-stress to the immature lung constitutes another approach to reduce the overall burden of BPD. Here, optimization of the noninvasive respiratory support with advanced modes of ventilation but particularly the noninvasive application of surfactant have been shown to have therapeutic potential [35,36,37,38]. Lastly, the avoidance of oxygen toxicity to the lung by reactive oxygen species formation by avoidance of hyperoxemic but as well hypoxemic episodes is another field of intensive research and the data on this topic derive the therapeutic potential of keeping the preterm infant more stable in the oxygen saturation target [39,40]. We recently added evidence to these two topics within our STENA cohort study: (1). rapid enteral feeding advances improved somatic growth for weight, length and head circumference until a corrected age of 36 weeks and (2). STENA did not impede successful stabilization on noninvasive respiratory support and the rate of infants requiring invasive mechanical ventilation decreased from 46% to 25% with the advances in clinical routine care over the total study period. Although the study was underpowered to detect statistically significant differences for the outcome bronchopulmonary dysplasia, the subgroup analysis of infants between 500 and 999 g revealed a trend towards reduced incidence of BPD in the fast advancement group with a *p*-value of 0.16 and of 0.152 for the most severely affected infants fulfilling the severe BPD criterion [41]. Here, we provide the 2-year follow-up data of our STENA cohort study on somatic growth and psychomotor outcomes.

## 2. Materials and Methods

### 2.1. Study Design

This study was designed as a retrospective follow-up of our recently published single-center observational study evaluating safety and short-term clinical outcome parameters after implementing a rapid standardized enteral nutritional advances (STENA) protocol in preterm infants <1500 g BW including daily milk increments of 20–30 mL/kg of body weight [41]. Originally, *n* = 363 infants were available for analysis, of which *n* = 70 were excluded (exclusion criteria contained major congenital malformations, severe syndromic diseases, or death before 36 weeks of gestation). Group sizes before and after implementation of STENA were nearly equivalent with *n* = 145 and *n* = 148 infants available for analysis respectively. Here, we provide the 2-year outcome data concerning somatic growth and psychomotor development. Ethical approval was obtained from the ethics committee of the Justus-Liebig-University of Giessen (Az. 98/2014) prior to the start of the analyses. All patient data were retrieved from the electronic patient management system as described previously. Baseline characteristics included birth weight and z-scores at delivery, gestational age, gender, multiple birth and provision of antenatal corticosteroids which was counted when at least one dosage was given 24 h prior to delivery (Table 1). SGA status was defined as birth weight <10th percentile according to the national reference values for our population [42,43]. Nutritional supply during the observation period and during the total duration in the neonatal intensive care unit (NICU) was provided as recommended by the ESPEGHAN committee [44]. All infants were supplied with the breast milk from their own mother. The criterion for inclusion into this follow-up investigation was complete documentation of the auxologic parameters plotted according to gender and corrected age (post term), completed neurodevelopmental evaluation using the Bayley Scales of Infant Development III, the gross motor function classification system (GMFCS) and evaluation of severe hearing impairment (need for hearing aids or cochlear implantation) or blindness (visual acuity <6/60 m) as requested within the follow-up by the Joint Federal Committee (GBA) in Germany [45,46,47]. All assessments were performed in a standardized manner by certified examiners trained to reliability that were blinded to the study aims and infant group allocation. After exclusion of patients lost to follow-up (*n* = 75, Table 2 and Table S1), we had 218 infants (74.4% of the original cohort) left in the study (Table 1). Analogous to the original cohort, the study collective was divided into three weight categories for a complementary subgroup analysis: BW ≤ 500 g (*n* = 11), 500 g < BW ≤ 1000 g (*n* = 102) and 1000 g < BW ≤ 1500 g (*n* = 105, Table S2).

### 2.2. Statistical Analysis

The absolute and relative frequencies of parameters are given for counted data. Comparisons were carried out using Wilcoxon rank-sum test for metric data and Pearson’s χ2 with continuity correction for categorical data. The demographics in Table 1 are shown as medians and interquartile ranges. Statistical analyses were performed using R, version 4.0.2 (R Foundation for Statistical Computing). There was no need to control for possible confounders because there were no differences regarding baseline demographic and perinatal characteristics (Table 1). Statistical significance was defined as a *p* value of < 0.05.

## 3. Results

### 3.1. Demographics

Compared with the total cohort, the infants successfully followed-up (*n* = 218) were smaller and more immature than those not available for analyses in both groups (*n* = 46, standard group BW *p* = 0.089 and GA *p* = 0.013, fast group *n* = 29, BW *p* = 0.003 and GA *p* = 0.001, Table 2). Even the proportion of SGA infants in the lost to follow-up collective was equal to the 2-year follow-up cohort (standard group *p* = 0.166, fast group *p* = 0.506). There was no need to control for possible confounders because there were no differences between infants followed-up in the pre-STENA and STENA implementation group regarding baseline demographics and perinatal characteristics including the outcomes determining parameters of birth weight, gestational age, SGA status, sex, multiple birth and provision of antenatal corticosteroids (Table 1). In the standard advancement group, median GA was 28.86 weeks (interquartile range [IQR] 26.57–30.93) and median BW 990 g (IQR 745–1385 g). In the fast advancement group, median GA was 28.71 weeks (IQR 26.43–30.43) and median BW 990 g (IQR 825–1340 g) with balanced gender distribution (41% vs. 53%, *p* = 0.092) and equal proportion of infants born small for gestational age (24% vs. 29%, *p* = 0.483). Frequency of multiples (33% vs. 46%, *p* = 0.501) and the use of antenatal corticosteroids (95% vs. 94%, *p* = 1.0) did not differ between both groups, as well as the z-scores for the auxologic parameters birth weight, length and head circumference at birth (Table 1).

### 3.2. Somatic and Psychomotor Outcome with 2 Years Corrected Age

Evaluating somatic growth, z-scores at 2 years corrected age were no longer in favor of rapid enteral feeding advances for weight (Δz-score median −0.74 vs. −0.53, *p* = 0.256, Table 3) and length (Δz-score median −0.54 vs. −0.45, *p* = 0.259, Table 3). But the difference for head circumference in favor of the fast group recently reported for the corrected gestational age of 36 weeks persisted until the age of 2 years (Δz-score median −0.94 vs. −0.55, *p* = 0.034, Table 3). The detailed z-score analyses attributed the difference of Δz-score head circumferences to the period until discharge while it remained unchanged from there until 2 years of age (Δz-score median 0.43 vs. 0.32, *p* = 0.827, Table 3).

With respect to the psychomotor outcome at 2 years corrected age, we found no statistically significant differences in the mental developmental index (MDI) (median score 95 vs. 95, *p* = 0.738) and in the psychomotor developmental index (PDI) (median score 109 vs. 103, *p* = 0.122) (Table 3). Furthermore, scores on GMFCS (median score 1 vs. 1, *p* = 0.170), proportion of infants with severe hearing impairment (median score 2% vs. 4%, *p* = 0.638) or blindness (median score 0% vs. 1%, *p* = 1.0) did not diverge significantly (Table 3). Separately considering data by the birthweight categories <500 g, 500–999 g and 1000–1499 g, the persistent improvement in head circumference was mostly applicable to the subgroup of 500 < BW ≤ 1000 g (standard group *n* = 47 vs. fast group *n* = 55, Δz-score median −0.78 vs. 0.43, *p* < 0.001) underlining the benefit particularly to this high-risk population (Table S2) while the number of infants studied in the <500 g birth weight strata was too low (standard group *n* = 4 vs. fast group *n* = 7) for a meaningful analysis (Table S2). As SGA infants are at risk for persistent growth deficits independent of the postnatal nutritional supply, we separately considered the somatic growth trajectories and psychomotor outcomes for nonSGA and SGA infants defined as birth weight below the 10th percentile. As can be expected from the published literature, auxiologic parameters for weight, length and head circumference at 2 years of age were significantly lower in SGA infants compared to nonSGA infants for both study groups. But when considering Δz-score medians as the appropriate readout to study somatic growth from birth until 2 years of age, no significant differences were detected for length and head circumference between SGA and nonSGA status while Δz-score median for weight was significantly higher in the SGA subgroup both before and after the intervention (Table 3). We furthermore compared SGA infants before and after implementation of STENA and no significant differences were detected for somatic growth and psychomotor outcomes but the number of infants in each group (*n* = 24 vs. *n* = 35) was quite low (Table 4 and Table 5).

**Figure 1:**
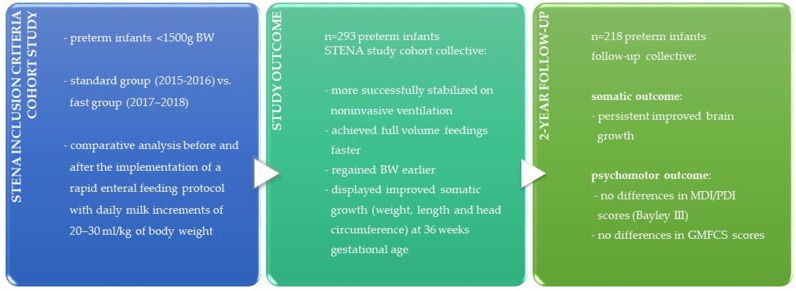
Summary of the main (significant) STENA cohort study results including the results from the previous publication on the short-term outcomes before discharge from the NICU [20].

All main results of our STENA cohort study on respiratory outcomes, somatic growth and psychomotor outcome are summarized in Figure 1 naming the short-term outcomes of our previous publication and the now presented follow-up data.

## 4. Discussion

### 4.1. Main Results

Our follow-up data adds important new insights on the compatibility of rapid enteral feeding advances and in parallel successful stabilization of preterm infants <1500 g on noninvasive respiratory support. Our results display that the effects of rapid enteral feeding advances on birth weight and body length were transient, but infants remained within the expected range defined as physiologic development with median z-scores not below −1.0. Of clinical importance, the persistent improved head circumference may hint towards improved brain growth although the data on the psychomotor outcomes did not reveal any advantage of STENA. Even if the results of our study suggest equal findings for psychomotor outcomes, we suggest to be careful in interpreting and drawing conclusions. We need to acknowledge, that the retrospective data collection, the restriction to 2-year follow-up outcomes and the nonavailability of nutritional supply data during follow-up prohibit more detailed insights into the relationship between nutritional supply and psychomotor outcomes. Although countless other factors influence preterm brain development, most notably the familiar educational and socioeconomic background, and these adversities can have substantial impact on the awareness of the important role of adequate nutritional supply with respect to the quantity but as well to the quality of nutrition that deserves particular attention during the first two years of life and undergoes several changes from exclusive milk feeding to table nutrition. The on average better somatic growth at discharge may led to less attention to further somatic growth or resulted in reduced total caloric intake, lower quality of nutritional supply including the duration of breast milk provision or less support of psychomotor development in the STENA group infants as these infants appeared more robust. Vice versa, the pre-STENA cohort could have been followed up more intensively due to the somatic growth deficits at discharge that resulted in catch-up of somatic growth parameters in weight and length until 2 years of age. In that direction, our results on Δz-score median for weight suggest variations in caloric intake between nonSGA and SGA infants but that did not result in catch-up growth for length and head circumference. The fact that our lost-to follow-up cohort was less immature and probably more stable after discharge probably made the families and pediatricians think that medical aftercare or final outcome testing was not required. This is also reflected by the data from the population-based prospective EPICE cohort study evaluating the routine follow-up of very preterm infants < 32 weeks of gestation [48]. Of all children (*n* = 3635), 90.3% had used follow-up services, and 27.3% were still doing so at 5 years of age. Never using follow-up services was associated with maternal sociodemographic characteristics (younger age, low educational level and being born outside Europe) and lower perinatal risk including higher GA (13.8% of infants born > 30 weeks GA never used specialized medical aftercare). Our follow-up data is in line with the results from the large multicenter SIFT-trial and supports their results of no significant impact of rapid enteral feeding advances on survival without moderate or severe neurodevelopmental disability at 24 months in very-low-birth-weight infants [26]. In this direction, the key features of aberrant preterm brain development go far beyond brain growth and include alterations in neuronal connectivity as one morphological correlate of impaired psychomotor development [49,50,51]. These key features were probably not affected by STENA that was intended towards more rapid enteral feeding advances while the total amount of macronutrient supply and quality of nutritional intake should not have been altered as nutritional supply was provided based on a standard protocol and all infants were fed with the breast milk from their own mother that could have impacted the outcomes.

Our focus on the birth weight subcategories and results are in line with the conclusion from the actual Cochrane meta-analysis which constituted that results of rapid enteral feeding advances are equally applicable to the categories <1000 g and 1000 to 1499 g of birth weight [25]. The trend towards better somatic growth is in line with the current state of relevant studies and the lack of statistical significance in our data can be caused by the relatively small sample size. Our results might even indicate that the most vulnerable population of extremely low birth weight infants <1000 g might mostly benefit from such an approach as in this population the risk of abnormal somatic development is particularly high. This is also underlined by another study the topic highlighting the need of GA-related feeding trajectories and monitoring of different feeding patterns for early identification of morbidities such as extrauterine growth restriction [52]. Furthermore, our data are clearly applicable to gold standard of actual nutritional recommendations as our population of preterm infants was at least partly provided with breast milk. In this respect, our results advance the recent meta-analyses for improved somatic growth outcomes and the total amount of breast milk intake in the first weeks of life might deserve further exploration [25,27]. In addition, our STENA approach of standardized rapid enteral feeding advances included instructions not to reduce the next bolus feeding when preset limits of pre-prandial gastric residuals of >5 mL/bodyweight in 50% of all daily feeds were not exceeded. Our results confirm the previous meta-analyses on this topic and add further data that rapid enteral feeding advances plus tolerating pre-prandial gastric residuals until preset limits are safe [28,29,41]. Lastly, our short-term outcomes demonstrated that rapid enteral feeding advances can be achieved together with successful stabilization on noninvasive respiratory support thereby avoiding mechanical ventilation. While a trend towards a benefit for the pulmonary outcome BPD was detected (subgroup analysis 500 < BW ≤ 1000 g, *p* = 0.160), the study was underpowered to detect significant effects on BPD but the association between better somatic growth and reduced risk for BPD was established within other observational studies on this topic and the data from our STENA cohort on somatic growth and BPD as well point into this direction [41]. Considering these results together with the now presented data on the psychomotor outcome at 2 years of age one might argue that avoiding shear stretch by mechanical ventilation is suited to reduce the trauma to the immature lung but cannot be hold responsible as cause for the abnormalities in psychomotor outcome in preterm infants. Here not even a trend towards a benefit was detectable.

### 4.2. Strengths and Limitations of the Study

One strength of our study is the high follow-up rate and the fact that we only had 25% of infants lost to follow-up. Furthermore, the two groups were highly comparable concerning their baseline demographics. That is why risk adjustment for confounders was not required. On the other hand, the limitations of our study need to be listed: Due to the retrospective nature of our study and the legal restrictions of follow-up only until the age of 2 years by the GBA in Germany we will not be able to provide later follow-up data and outcomes on higher-level skills that cannot be tested at the age of 2 years. However, these functional impairments constitute the predominant limitations of psychomotor function in former preterm infants that outreach gross motor function and mental development at 2 years of age. As parental auxiologic parameters were not recorded during clinical routine, we cannot describe potential variations between our two groups that might have impacted the outcome for head circumference. Furthermore, we cannot exclude carry-over effects between one and the other medical measure. From the large multicenter study COT [53] it is known that prolonged hypoxemias and bradycardias are associated with worse psychomotor outcome. We did not monitor whether more hypoxemias or bradycardias occurred in the STENA group that was highly successfully stabilized on noninvasive ventilation (NIV) compared to the standard group (54% vs. 75%) [41]. The intention to avoid mechanical ventilation might have changed the attitudes of the clinical team towards tolerating more hypoxemic events and subsequent hyperoxemias and higher fractions of oxygen prior to considering intubation with a higher overall ROS burden during NIV in the STENA cohort than in the pre-STENA group. Lastly, our short-term outcomes demonstrated that rapid enteral feeding advances can be achieved together with successful stabilization on noninvasive respiratory support thereby avoiding mechanical ventilation. While a trend towards a benefit for the pulmonary outcome BPD was detected (subgroup analysis 500 < BW ≤ 1000 g, *p* = 0.160), the study was underpowered to detect significant effects on BPD but the association between better somatic growth and reduced risk for BPD was established within other observational studies on this topic and the data from our STENA cohort on somatic growth and BPD as well point into this direction [41]. Considering these results together with the now presented data on the psychomotor outcome at 2 years of age one might argue that avoiding shear stretch by mechanical ventilation is suited to reduce the trauma to the immature lung but cannot be hold responsible as cause for the abnormalities in psychomotor outcome in preterm infants. Here not even a trend towards a benefit was detectable.

## 5. Conclusions

In conclusion, our follow-up results are in line with the previous observations on this topic [27] and confirm the safety of rapid enteral feeding advances with respect to somatic growth and the psychomotor outcome. It needs to be recognized that one isolated intervention will probably not alter the overall outcome that is impacted by a multitude of risk factors or the changes are too marginal to be detected within the restricted number of patients studied. However, regimes of rapid enteral feeding advances particularly in the context of tolerating pre-prandial gastric residuals together with strategies to avoid mechanical ventilation deserve further exploration with particular focus on the psychomotor and pulmonary outcome. Our study results underline the potential for future studies on this topic in order to limit growth retardation and adverse neurologic outcome. And it highlights the need for further evidence-based studies that include the longer-term outcomes in VLBWI into their study plan.

## Figures and Tables

**Table 1:** Demographics and infant characteristics of the STENA cohort study followed-up at 24 months.

	2-Year Follow-Up Standard Group (2015–2016) *n* = 99	2-Year Follow-Up Fast Group (2017–2018) *n* = 119	*p*-Value
Birth weight, g	990 (745–1385)	990 (825–1340)	0.994 ^a^
Gestational age, weeks	28.86 (26.57–30.93)	28.71 (26.43–30.43)	0.839 ^a^
z-scores at birth			
weight	−0.62 (−1.18–−0.16)	−0.65 (−1.38–−0.13)	0.662 ^a^
length	−0.61 (−1.09–−0.29)	−0.59 (−1.17–−0.19)	0.600 ^a^
head circumference	−0.72 (−1.20–−0.27)	−0.80 (−1.24–−0.27)	0.915 ^a^
SGA ^c^, *n* (%)	24 (24)	35 (29)	0.483 ^b^
Male sex, *n* (%)	41 (41)	64 (53)	0.092 ^b^
Multiple birth, *n* (%)	33 (33)	46 (39)	0.501 ^b^
Antenatal corticosteroids, *n* (%)	94 (95)	112 (94)	1.000 ^b^

Note: Data shown as median (interquartile range) or *n* (%). ^a^ Wilcoxon test, ^b^ Pearson with Yates’ continuity correction test, ^c^ small for gestational age.

**Table 2:** Demographics and characteristics of the lost to follow-up collective vs. follow-up collective.

	2-Year Follow-Up *n* = 218	Lost to Follow-Up *n* = 75	*p*-Value
**Standard group (2015–2016)**	*n* = 99	*n* = 46	
Birth weight, g	990 (745–1385)	1175 (910–1395)	0.089 ^a^
Gestational age, weeks	28.86 (26.57–30.93)	30.21 (27.86–31.71)	0.013 ^a^
z-Scores at birth			
weight	−0.62 (−1.18–−0.16)	−0.89 (−1.44–−0.03)	0.376 ^a^
length	−0.61 (−1.09–−0.29)	−0.59 (−1.05–−0.03)	0.833 ^a^
head circumference	−0.72 (−1.20–−0.27)	−0.79 (−1.23–−0.38)	0.441 ^a^
SGA ^c^, *n* (%)	24 (24)	17 (37)	0.166 ^b^
Male sex, *n* (%)	41 (41)	27 (59)	0.078 ^b^
Multiple birth, *n* (%)	33 (33)	27 (59)	0.007 ^b^
Antenatal corticosteroids, *n* (%)	94 (95)	40 (87)	0.075 ^b^
**Fast group (2017–2018)**	*n* = 119	*n* = 29	
Birth weight, g	990 (825–1.340)	1.320 (995–1.440)	0.003 ^a^
Gestational age, weeks	28.71 (26.43–30.43)	30.43 (28.14–32.86)	0.001 ^a^
z-Scores at birth			
weight	−0.65 (−1.38–−0.13)	−1.07 (−1.65–−0.32)	0.158 ^a^
length	−0.59 (−1.17–−0.19)	−0.66 (−1.38–−0.25)	0.504 ^a^
head circumference	−0.80 (−1.24–−0.27)	−0.90 (−1.19–−0.37)	0.623 ^a^
SGA ^c^_,_ *n* (%)	35 (29)	11 (38)	0.506 ^b^
Male sex, *n* (%)	64 (53)	64 (59)	0.794 ^b^
Multiple birth, *n* (%)	46 (39)	12 (41)	0.954 ^b^
Antenatal corticosteroids, *n* (%)	112 (94)	25 (86)	0.209 ^b^

Note: Data shown as median (interquartile range) or *n* (%). ^a^ Wilcoxon test, ^b^ Pearson with Yates’ continuity correction test, ^c^ small for gestational age.

**Table 3:** 2-year somatic and psychomotor outcome variables in the study collective.

	2-Year Follow-Up Standard Group (2015–2016) *n* = 99	2-Year Follow-Up Fast Group (2017–2018) *n* = 119	*p*-Value
z-Scores at 2 years corrected age			
weight	−0.74 (−1.43–−0.05)	−0.53 (−1.24–0.09)	0.256 ^a^
length head circumference	−0.54 (−1.39–0.19) −0.94 (−2.15–−0.10)	−0.45 (−1.31–0.47) −0.55 (−1.38–0.20)	0.259 ^a^ 0.034 ^a^
Δz-score (weight 2 years-birth)	0.07 (−0.79–0.61)	0.07 (−0.54–0.80)	0.243 ^a^
Δz-score (weight 2 years-36 weeks gestational age) Δz-score (length 2 years-birth) Δz-score (length 2 years-36 weeks gestational age) Δz-score (head 2 years-birth) Δz-score (head 2 years-36 weeks gestational age)	0.49 (−0.29–1.16) −0.01 (−0.68–0.65) 1.21 (0.25–1.83) −0.40 (−1.15–0.58) 0.43 (−0.80–1.08)	0.39 (−0.23–0.98) 0.26 (−0.75–0.97) 0.93 (−0.13–1.77) 0.26 (−0.73–1.15) 0.32 (−0.47–1.07)	0.591 ^a^ 0.291 ^a^ 0.183 ^a^ 0.007 ^a^ 0.827 ^a^
MDI ^c^	95 (85–105)	95 (80–105)	0.738 ^a^
PDI ^d^	109 (89–125)	103 (85–119)	0.122 ^a^
GMFCS ^e^	1 (1-1)	1 (1-1)	0.170 ^a^
Severe hearing impairment, *n* (%)	2 (2)	5 (4)	0.638 ^b^
Blindness, *n* (%)	0 (0)	1 (1)	1.000 ^b^

Note: Data shown as median (interquartile range) or *n* (%). ^a^ Wilcoxon test, ^b^ Pearson with Yates’ continuity correction test, ^c^ mental developmental index, ^d^ psychomotor developmental index, ^e^ Gross Motor Functions Classification System.

**Table 4:** 2-year somatic and psychomotor outcome variables in the study collective—SGA vs. nonSGA subgroup.

	2-Year Follow-Up SGA Subgroup *n* = 59	2-Year Follow-Up NonSGA Subgroup *n* = 159	*p*-Value
**Standard group (2015–2016)** z-Scores at 2 years corrected age	*n* = 24	*n* = 75	
weight	−1.18 (−1.91–−0.81)	−0.61 (−1.26–0.05)	0.012 ^a^
length head circumference	−1.54 (−2.17–−0.95) −2.04 (−3.35–−0.80)	−0.43 (−1.10–0.30) −0.74 (−1.61–0.04)	<0.001 ^a^ 0.004 ^a^
Δz-score (weight 2 years-birth)	0.48 (0.04–1.10)	−0.14 (0.80–0.42)	0.008 ^a^
Δz-score (weight 2 years-36 weeks gestational age) Δz-score (length 2 years-birth) Δz-score (length 2 years-36 weeks gestational age) Δz-score (head 2 years-birth) Δz-score (head 2 years-36 weeks gestational age)	0.52 (−0.07–1.62) 0.03 (−0.63–0.57) 1.46 (0.72–2.16) −0.81 (−1.44–0.57) −0.13 (−1.19–0.63)	0.44 (−0.40–1.10) −0.05 (−0.68–0.66) 1.12 (0.22–1.75) −0.29 (−1.04–0.55) 0.55 (−0.75–1.19)	0.213 ^a^ 0.978 ^a^ 0.171 ^a^ 0.384 ^a^ 0.129 ^a^
MD I ^c^	95 (85–105)	85 (81–105)	0.844 ^a^
PDI ^d^	101 (82–122)	111 (95–126)	0.247 ^a^
GMFCS ^e^	1 (1-1)	1 (1-1)	0.500 ^a^
Severe hearing impairment, *n* (%)	1 (4)	1 (1)	0.950 ^b^
Blindness, *n* (%)	0 (0)	0 (0)	-
**Fast group (2017–2018)** z-scores at 2 years corrected age	*n* = 35	*n* = 84	
weight	−1.12 (−1.69–−0.19)	−0.27 (−0.90–0.18)	0.005 ^a^
length head circumference	−0.90 (−1.43–−0.32) −1.12 (−2.14–−0.30)	−0.36 (−1.23–0.57) −0.26 (−1.07–0.25)	0.030 ^a^ 0.012 ^a^
Δz-score (weight 2 years-birth)	0.72 (0.06–1.58)	−0.17 (−0.72–0.60)	<0.001 ^a^
Δz-score (weight 2 years-36 weeks gestational age) Δz-score (length 2 years-birth) Δz-score (length 2 years-36 weeks gestational age) Δz-score (head 2 years-birth) Δz-score (head 2 years-36 weeks gestational age)	0.64 (0.01–1.42) 0.42 (−0.33–1.01) 1.01 (0.20–2.17) 0.15 (−1.20–1.13) 0.37 (−0.33–1.07)	0.27 (−0.24–0.91) 0.24 (−0.87–0.94) 0.81 (−0.19–1.72) 0.28 (−0.59–1.16) 0.32 (−0.47–1.07)	0.112 ^a^ 0.407 ^a^ 0.345 ^a^ 0.700 ^a^ 0.967 ^a^
MDI ^c^	85 (79–105)	95 (85–106)	0.198 ^a^
PDI ^d^	96 (85–116)	103 (87–120)	0.310 ^a^
GMFCS ^e^	1 (1-1)	1 (1-1)	1.000 ^a^
Severe hearing impairment, *n* (%)	1 (3)	4 (5)	1.000 ^b^
Blindness, *n* (%)	1 (3)	0 (0)	0.650 ^b^

Note: Data shown as median (interquartile range) or n (%). ^a^ Wilcoxon test, ^b^ Pearson with Yates’ continuity correction test, ^c^ mental developmental index, ^d^ psychomotor developmental index, ^e^ Gross Motor Functions Classification System.

**Table 5:** 2-year somatic and psychomotor outcome variables in the study collective—SGA subgroup.

	2-Year Follow-Up Standard Group (2015–2016) *n* = 24	2-Year Follow-Up Fast Group (2017–2018) *n* = 35	*p*-Value
z-Scores at 2 years corrected age			
weight	−1.18 (−1.91–−0.81)	−1.12 (−1.69–−0.19)	0.511 ^a^
length head circumference	−1.54 (−2.17–−0.95) −2.04 (−3.35–−0.80)	−0.90 (−1.43–−0.32) −1.12 (−2.14–−0.30)	0.079 ^a^ 0.114 ^a^
Δz-score (weight 2 years-birth)	0.48 (0.04–1.10)	0.72 (0.06–1.58)	0.457 ^a^
Δz-score (weight 2 years-36 weeks gestational age) Δz-score (length 2 years-birth) Δz-score (length 2 years-36 weeks gestational age) Δz-score (head 2 years-birth) Δz-score (head 2 years-36 weeks gestational age)	0.52 (−0.07–1.62) 0.03 (−0.63–0.57) 1.46 (0.72–2.16) −0.81 (−1.44–0.57) −0.13 (−1.19–0.63)	0.64 (0.01–1.42) 0.42 (−0.33–1.01) 1.01 (0.20–2.17) 0.15 (−1.20–1.13) 0.37 (−0.33–1.07)	0.955 ^a^ 0.315 ^a^ 0.302 ^a^ 0.213 ^a^ 0.265 ^a^
MDI ^c^	95 (85–105)	85 (79–105)	0.224 ^a^
PDI ^d^	101 (82–122)	96 (85–116)	0.747 ^a^
GMFCS ^e^	1 (1-1)	1 (1-1)	0.377 ^a^
Severe hearing impairment, *n* (%)	1 (4)	1 (3)	1.000 ^b^
Blindness, *n* (%)	0 (0)	1 (3)	1.000 ^b^

Note: Data shown as median (interquartile range) or *n* (%). ^a^ Wilcoxon test, ^b^ Pearson with Yates’ continuity correction test, ^c^ mental developmental index, ^d^ psychomotor developmental index, ^e^ Gross Motor Functions Classification System.

## Data Availability

The data that support the findings of this study are available on reasonable request from the corresponding author.

## Supplementary Materials

The following supporting information can be downloaded at: https://www.mdpi.com/article/10.3390/nu15051292/s1, Table S1: Demographics and characteristics of infants lost to follow-up of the STENA cohort study; Table S2: Subgroup analyses of infants followed-up at 2 years separated for birth weight (BW) categories.
