# Supplementary material for: Noninvasive Ventilation and Rapid Enteral Feeding Advances in Preterm Infants—2-Year Follow-Up of the STENA-Cohort
Source: Nutrients. 2023 Mar 6;15(5):1292. doi: 10.3390/nu15051292 (PMC10005522; doi:10.3390/nu15051292)
Supplement: Supplementary file 1 [file nutrients-15-01292-s001.zip › nutrients-2232965-supplementary.pdf]

Supporting information S1

E-Table S1: Demographics and characteristics of infants lost to follow-up of the STENA cohort study

	**lost to follow-up Standard group (2015-2016)** n = 46	**lost to follow-up Fast group (2017-2018)** n = 29	p-value
Birth weight, g	1175 (910-1395)	1320 (995-1440)	0.168[a]
Gestational age, weeks	30.21 (27.86-31.71)	30.43 (28.14-32.86)	0.215[a]
z-scores at birth			
weight	-0.89 (-1.44--0.02)	-1.07 (-1.65--0.32)	0.397[a]
length	-0.59 (-1.05--0.02)	-0.66 (-1.38--0.25)	0.636[a]
head circumference	-0.79 (-1.23--0.38)	-0.90 (-1.19--0.37)	0.964[a]
SGA[c]	17 (37)	11 (37)	1.000[b]
Male sex, n (%)	27 (59)	64 (59)	1.000[b]
Multiple birth, n (%)	27 (59)	12 (41)	0.221[b]
Antenatal corticosteroids, n (%)	40 (87)	25 (86)	0.428[b]

Note: Data shown as median (interquartile range) or n (%)
[a]Wilcoxon test
[b]Pearson with Yates' continuity correction test
[c]small for gestational age

E-Table S2: Subgroup analyses of infants followed-up at 2 years separated for birth weight (BW) categories

	**Standard group**<br>**(2015-2016)**<br>**n=99**<br><=500g BW<br>n=4<br>500 < BW <=1000g<br>n=47<br>1000 < BW <=1500g<br>n=48	**Fast group**<br>**(2017-2018)**<br>**n=119**<br><=500g BW<br>n=7<br>500 < BW <=1000g<br>n=55<br>1000 < BW <=1500g<br>n=57	**p-value**
**MDI**[c]			
<= 500g BW	80 (74-88)	80 (65-98)	0.930[a]
500 < BW <=1000g	95 (80-100)	95 (85-108)	0.486[a]
1000 < BW <=1500g	100 (90-110)	93 (80-105)	0.270[a]
**PDI**[d]			
<= 500g BW	76 (76-78)	82 (78-101)	0.330[a]
500 < BW <=1000g	102 (84-117)	100 (82-113)	0.724[a]
1000 < BW <=1500g	119 (103-128)	103 (96-127)	0.080[a]
**GMFCS**[e]			
<= 500g BW	2 (1-2)	1 (1-2)	0.598[a]
500 < BW <=1000g	1 (1-1)	1 (1-1)	0.034[a]
1000 < BW <=1500g	1 (1-1)	1 (1-1)	0.665[a]

**severe hearing impairment**			
<= 500g BW	0 (0)	0 (0)	-
500 < BW <=1000g	0 (0)	3 (5)	0.300[b]
1000 < BW <=1500g	2 (4)	2 (4)	1.000[b]
**blindness**			
<= 500g BW	0 (0)	1 (14)	1.000[b]
500 < BW <=1000g	0 (0)	0 (0)	-
1000 < BW <=1500g	0 (0)	0 (0)	-
**z-scores for auxologic parameters at 2 years corrected age**			
weight			
<= 500g BW	-1.13 (-1.44--1.09)	-0.89 (-1.85-0.64)	0.599[a]
500 < BW <=1000g	-0.83 (-1.43--0.35)	-0.53 (-1.48-0.15)	0.305[a]
1000 < BW <=1500g	-0.55 (-1.32-0.19)	-0.42 (-0.94-0.06)	0.632[a]
length			
<= 500g BW	-2.07 (-2.19--1.64)	-0.91 (-1.34--0.51)	0.230[a]
500 < BW <=1000g	-0.48 (-1.39-0.28)	-0.84 (-1.54-0.40)	0.985[a]
1000 < BW <=1500g	-0.54 (-1.29-0.09)	-0.40 (-0.99-0.57)	0.159[a]
head circumference			
<= 500g BW	-3.28 (-3.48--1.80)	-2.09 (-2.65--0.55)	0.458[a]
500 < BW <=1000g	-1.34 (-2.56--0.51)	-0.50 (-1.15-0.24)	0.004[a]
1000 < BW <=1500g	-0.50 (-1.49-0.24)	-0.51 (-1.19-0.09)	0.991[a]
**Δz-score (weight 2 years-birth)**			
<= 500g BW	0.45 (-0.04-0.92)	0.88 (0.67-1.28)	0.334[a]

500 < BW <=1000g	-0.24 (-1.00-0.32)	-0.06 (-0.69-0.60)	0.418[a]
1000 < BW <=1500g	0.19 (-0.60-0.89)	0.18 (-0.54-1.02)	0.787[a]
**Δz-score (weight 2 years-36 weeks GA)**			
<= 500g BW	-0.14 (-0.33-0.17)	0.62 (0.24-0.91)	0.147[a]
500 < BW <=1000g	0.38 (-0.37-0.90)	0.18 (-0.48-0.71)	0.349[a]
1000 < BW <=1500g	0.61 (-0.12-1.46)	0.62 (-0.11-1.33)	0.750[a]
**Δz-score (length 2 years-birth)**			
<= 500g BW	-0.33 (-0.42--0.29)	-0.54 (-1.09-0.11)	0.689[a]
500 < BW <=1000g	-0.11 (-0.78-0.62)	-0.16 (-0.89-0.64)	0.887[a]
1000 < BW <=1500g	0.09 (-0.67-0.72)	0.64 (-0.49-1.28)	0.093[a]
**Δz-score (length 2 years-36 weeks GA)**			
<= 500g BW	-0.06 (-0.09-0.66)	1.01 (0.11-2.73)	0.599[a]
500 < BW <=1000g	1.43 (0.61-2.19)	0.62 (-0.23-1.61)	0.012[a]
1000 < BW <=1500g	0.86 (0.16-1.52)	1.05 (0.09-1.81)	0.725[a]
**Δz-score (head 2 years-birth)**			
<= 500g BW	-0.76 (-1.55-0.06)	-0.76 (-1.65-0.99)	0.689[a]
500 < BW <=1000g	-0.78 (-1.77-0.13)	0.43 (-0.38-1.32)	<0.001[a]
1000 < BW <=1500g	0.04 (-0.94-0.85)	0.06 (-0.63-1.02)	0.736[a]
**Δz-score (head 2 years-36 weeks GA)**			
<= 500g BW	-1.22 (-1.41--0.21)	0.20 (-0.48-0.93)	0.334[a]
500 < BW <=1000g	0.04 (-1.24-0.98)	0.51 (-0.53-1.25)	0.277[a]
1000 < BW <=1500g	0.55 (-0.27-1.23)	0.29 (-0.45-0.79)	0.265[a]

Note: Data shown as median (interquartile range) or n (%)

[a]Wilcoxon test

[b]Pearson with Yates' continuity correction test
